# Alere Determine-tuberculosis lipoarabinomannan positivity in disseminated non-tuberculous mycobacteria: An illustrative case series

**DOI:** 10.4102/sajhivmed.v23i1.1369

**Published:** 2022-04-04

**Authors:** Riana Greyling, Graeme Meintjes, Bianca Sossen

**Affiliations:** 1 Matthew Goniwe Clinic, Cape Town City Health Department, Cape Town, South Africa; 2 Department of Medicine, Faculty of Health Sciences, University of Cape Town, Cape Town, South Africa; 3 Wellcome Center for Infectious Diseases Research in Africa, Institute of Infectious Disease and Molecular Medicine, University of Cape Town, Cape Town, South Africa

**Keywords:** tuberculosis, Lipoarabinomannan, ambulatory, outpatient, point-of-care, urine, HIV, diagnostic

## Abstract

**Introduction:**

In outpatients, the World Health Organization recommends that the urine Alere Determine-tuberculosis lipoarabinomannan (AlereLAM) should be used to support the diagnosis of tuberculosis (TB) in people living with HIV (PLHIV) with CD4 counts ≤ 100 cells/µL or with signs of being ‘seriously ill’. There is a risk of a false-positive AlereLAM in disseminated non-tuberculous mycobacterial (NTM) infections and it may be difficult to differentiate a single infection (either *Mycobacterium tuberculosis* or NTM) from dual infection.

**Patient presentation:**

We report three patients, enrolled in an operational study assessing AlereLAM use in an outpatient setting, who had advanced HIV (all CD4 < 20 cells/µL) and strongly positive (grade 4+) AlereLAM results in whom Mycobacterium avium or kansasii were later cultured from blood or urine and sputum.

**Management and outcome:**

Based on positive AlereLAM results, all three were initiated on TB treatment. One died before NTM infection was detected. Two were managed for dual infection (TB and NTM) but died within two years.

**Conclusion:**

Tuberculosis remains a leading cause of death and a disproportionate number of these deaths occur in PLHIV. Tuberculous treatment should be initiated based on a positive AlereLAM result, and this should be followed by additional testing to confirm the diagnosis of TB and to obtain drug susceptibility results. In those not responding to TB treatment where the only positive result was an AlereLAM, an alternative or additional diagnosis of NTM infection should be considered, particularly in patients with a very low CD4 count.

## Introduction

The World Health Organization (WHO) recommends the use of the Alere Determine-tuberculosis lipoarabinomannan assay, or AlereLAM, in outpatient settings for people living with HIV (PLHIV) with CD4 counts ≤ 100 cells/µL or signs of being ‘seriously ill’.^1^ Evidence in support of this includes improved survival when AlereLAM is implemented in hospitalised, ill PLHIV, alongside its low cost, rapid turnaround time and instrument-free design.^1,2,3^ The diagnostic sensitivity and specificity of AlereLAM in outpatient settings are estimated at 31% and 95%.^4^

Lipoarabinomannan is present in the cell wall of all mycobacterium species. Different LAM assays have been designed to detect different LAM domains, some more specific to *Mycobacterium tuberculosis* (*M. tb*) than others.^5^ There is a risk of false-positive AlereLAM results in the setting of disseminated non-tuberculous mycobacterial (NTM) infections and it may be difficult to differentiate a single infection (either *M. tb* or NTM) from a dual infection.

With the ongoing scale-up of AlereLAM use in the South African public health sector, awareness of the possibility of a false-positive AlereLAM due to NTM infection is important. This is particularly critical as the patient groups affected by disseminated *M. tb* and NTM infections (i.e. low CD4 counts) overlap.^6^ To highlight this, we report three patients who were enrolled in a study of AlereLAM utility at outpatient clinics in Khayelitsha, Cape Town.^7^

## Case reports

### Patient 1

An antiretroviral therapy (ART)-naive woman was asymptomatic for tuberculosis (TB) at presentation and started on first-line ART (see Table 1 for more information). She rapidly deteriorated and presented again after 10 days with night sweats, a cough, and abdominal pain. She met the criteria for AlereLAM testing on this date, which revealed a strongly positive (4+) result. The patient was placed onto TB treatment and sputum was sent for Xpert MTB/RIF Ultra and mycobacterial culture, along with urine for mycobacterial culture.

**TABLE 1:** Clinical characteristics of three patients.

Clinical feature	Patient 1	Patient 2	Patient 3
Demographic profile	41-year-old woman	37-year-old man	33-year-old man
HIV diagnosis status	Known to be living with HIV	Known to be living with HIV	Newly diagnosed HIV
ART status	ART-naive	Second-line ART	Recently started first-line ART
CD4 count (cells/μL)	11	7	13
TB status	History of TB 10 years prior. Negative WHO TB symptom screen.	History of TB four times prior and presented with TB symptoms.	No known history of TB. Negative WHO TB symptom screen.
AlereLAM result	Positive Grade: 4+	Positive Grade: 4+	Positive Grade: 4+
Mycobacterial testing	Sputum: NTM† Urine: NTM† Blood: *M. avium*	Sputum: *M. kansasii* Urine: *M. kansasii*	Blood: *M. avium*
Treatment received‡	RHZE	RHZE followed by RHZE + Azithromycin	RHZE followed by RHE + Azithromycin
Outcome	Died 3 months after presentation	Died 24 months after presentation	Died 17 months after presentation

AlereLAM, Alere Determine-tuberculosis lipoarabinomannan assay; ART, antiretroviral therapy; NTM, non-tuberculous mycobacteria; TB, tuberculosis; WHO, World Health Organization.

† , No further identification was conducted by laboratory;

‡ , R = Rifampicin; H = Isoniazid; Z = Pyrazinamide; E = Ethambutol.

After one month of TB treatment, the patient’s condition worsened. She presented with vomiting and abdominal distension and was referred for inpatient care. By this time the sputum and urine cultures had detected NTM without further identification and a mycobacterial blood culture was then drawn. The patient died during hospital admission. After her death, the laboratory reported that the blood culture identified *Mycobacterium avium*. No Xpert MTB/RIF or cultures identified *M. tb*.

### Patient 2

A man who was on second-line ART and had a history of TB four times prior presented with weight loss, fatigue and diffuse, hypopigmented skin lesions suggestive of a superficial fungal infection (see Table 1 for more information). The patient was classified as meeting the WHO ‘seriously ill’ criteria and an AlereLAM revealed a strongly positive (4+) result; he was therefore started on TB treatment. A sputum and urine sample were collected, and both of these later cultured *Mycobacterium kansasii*. Once the two samples had isolated *M. kansasii*, a dual infection of *M. tb* and *M. kansasii* was diagnosed and azithromycin was added to the patient’s regimen. Unfortunately, the patient disengaged from treatment after one year and died as an inpatient two years after initial presentation. Blood culture on that admission was positive for *Mycobacterium kansasii*. No Xpert MTB/RIF or cultures identified *M. tb.*

### Patient 3

A man with recently diagnosed HIV and cryptococcal meningitis had been started on ART one month prior to this (see Table 1 for more information). He presented with WHO ‘seriously ill’ criteria and an AlereLAM was performed, revealing a strongly positive (4+) result. He was started on TB treatment on the same day. A month later, he deteriorated clinically and required hospital admission. A blood culture during this admission cultured *Mycobacterium avium*. With this result, azithromycin was added to his TB treatment and pyrazinamide was stopped. The patient died as an inpatient 17 months after initial presentation. No Xpert MTB/RIF or cultures identified *M. tb,* although he was diagnosed with TB meningitis based on CT brain features during the protracted illness course.

## Discussion

A disproportionate number of tuberculosis deaths occur in PLHIV.^8^ Tuberculosis treatment should be initiated based on a positive AlereLAM result,^1^ so that ill patients who are at high risk of dying will benefit from the test and a positive result.

These case reports illustrate that a positive AlereLAM result must be interpreted in the clinical context and further diagnostic testing should be conducted. The objectives of further investigations are mycobacterial species identification and drug susceptibility testing. If a patient with advanced immunosuppression is initiated on TB treatment based on an AlereLAM alone and further sampling is not possible, it is important to monitor the patient’s response to tuberculosis treatment closely. In the case of a non-response to treatment or deterioration on treatment, NTM infection or dual infection (with *M. tb* and NTM) should be considered, particularly in patients with very low CD4 counts, together with other differential diagnoses. Finally, these case reports illustrate that disseminated NTM infections can result in strongly positive AlereLAM results. Whether this finding can be used as an indicator of disseminated NTM infection needs to be assessed further in larger studies.
